# Sleep promotion for hospitalised children: Developing an evidence-based guideline for nurses

**DOI:** 10.4102/curationis.v44i1.2219

**Published:** 2021-10-06

**Authors:** Elijeshca C. Crous, Natasha North

**Affiliations:** 1 The Harry Crossley Children’s Nursing Development Unit, Department of Paediatrics and Child Health, Faculty of Health Sciences, University of Cape Town, Cape Town, South Africa

**Keywords:** sleep, hospital, children, paediatric, nursing, guideline

## Abstract

**Background:**

Adequate sleep in hospitalised children is important for a variety of physiological and psychological processes associated with growth, development, and recovery from illness and injury. Hospitalisation often prioritises clinical care activities at the expense of age-appropriate sleep. Nurses and the wider healthcare team contribute to this paradox. However, through conscious practice and partnering with mothers, nurses are able to enact change and promote sleep.

**Objectives:**

To adopt, adapt or contextualise existing guidelines to develop an evidence-based practice guideline to promote sleep-friendly ward environments and routines facilitated by nurses, and in partnership with mothers.

**Method:**

A six-step methodology for guideline adaptation was followed, as recommended by the South African Guidelines Excellence project: (1) existing guidelines and protocols were identified and (2) appraised using the AGREE II instrument; (3) an evidence base was developed; (4) recommendations were modified, (5) assigned levels of evidence and grades of recommendation; and (6) end user guidance was developed. Expert consultation was sought throughout.

**Results:**

Existing relevant guidance comprised 61 adult-centric recommendations. Modification of the evidence base led to six composited recommendations that facilitate sleep in hospitalised children: (1) prioritising patient safety; (2) collaborating with the mother or caregiver to promote sleep; (3) coordinating ward routine and (4) environment to improve sleep; (5) work with clinical and non-clinical staff; and (6) performing basic sleep assessments. Practice recommendations were aligned to the South African regulatory framework for nursing.

**Conclusion:**

Hospitalisation is a time of physiological and psychological dysregulation for children, which is amplified by poor sleep in a hospital. Nurses have the opportunity to promote sleep during hospitalisation by implementing this African-centric guideline in partnership with mothers.

## Introduction

Sleep is crucial to the developmental processes of children (Beebe 2011; Davis, Parker & Montgomery 2004; Grigg-Damberger 2016), and is important in regulating emotion, behaviour, coping and perceptions of pain (Bevan et al. 2019; Jimenez et al. 2018; Stickland et al. 2016). Sleep regulates vital signs (Dennis et al. 2010), improves immunity and supports recovery from illness and injury (DeKeyser Ganz 2012; Gamaldo, Shaikh & McArthur 2012). Ill-health, some medications and pre-existing sleep dysfunctions all have a negative impact on the duration and quality of sleep of patients in hospitals (Herbert et al. 2014; Morse & Bender 2019).

In critically ill adults, length of stay is adversely affected by in-hospital sleep disruption (Morse & Bender 2019). Sleep in hospitalised children is disrupted by an unfavourable environment that prioritises disease management above sleep (Bevan et al. 2019; Setoyama, Ikeda & Kamibeppu 2016; Stickland et al. 2016), evidenced by a quantitative loss of more than one hour sleep per night during hospitalisation (Bevan et al. 2019). Pharmacological strategies to improve sleep have disadvantages, including the potential for unwanted daytime sedation, added cost and medication errors.

Rigorously developed non-pharmacological interventions for sleep promotion are not well represented in the literature: a systematic review on non-pharmacological sleep promotion in hospitalised children is underway (Kudchadkar et al. 2017). A study on sleep promotion in Africa focused on the skin-to-skin contact and sleep in 16 full-term neonates (Morgan, Horn & Bergman 2011), but no studies have been identified which focus on non-pharmacological interventions for older babies and children in African settings.

Non-pharmacological sleep promotion is a safer and more holistic alternative to pharmacological sleep promotion which is actionable by nurses. Nurses contribute to sleep disruption in hospitals (Monsén & Edéll-Gustafsson 2005), but are also positioned to improve sleep through advocacy, practice change (Meltzer, Davis & Mindell 2012) and by capitalising on unique relational strengths (Keys & Benzies 2018). The centrality of forming therapeutic relationships and creating a therapeutic environment that aids the patient’s well-being is recognised as a core part of the nurses’ role (South African Nursing Council [SANC] 2013:786). Nurses partnering with families in hospital hold the additional potential for improved sleep habits to extend beyond the period of hospitalisation, benefiting the child’s long-term health trajectory (Erondu et al. 2019).

To address variable in hospital sleep practices, an evidence-based practice guideline (EBPG) was created. A guideline is a rigorously developed document that informs healthcare decisions through actionable recommendations (Dizon, Machingaidze & Grimmer 2016; Rosenfeld, Shiffman & Robertson 2013). The underlying rigour of guidelines assists with standardising and improving the quality of care (Davino-Ramaya et al. 2012; Liang et al. 2017; Rosenfeld et al. 2013).

### Aim and purpose

The aim of this study was to adopt, adapt or contextualise existing guidelines to develop an evidence-based practice guideline to promote sleep-friendly ward environments and routines facilitated by nurses, and in partnership with mothers.

The central purpose of developing the new guideline is to provide evidence-based recommendations to assist nurses in optimising the quantity and quality of sleep in hospitalised children, contributing to the longer-term goal of aiding healing and emotional well-being. This article describes the process of development. Recommendations are presented together with a flowchart summary (see Figure 4) to aid implementation.

## Research methods and design

### Overview of study design

In order to promote sleep in hospitalised children, a six-step methodology was followed: (1) existing guidelines and protocols were identified and (2) appraised using the AGREE II instrument; (3) an evidence base was developed; (4) recommendations were modified and (5) assigned levels of evidence and grades of recommendation after which (6) end user guidance was developed. Expert consultation was sought throughout the process, particularly during the fourth and sixth stages of development. This methodology for guideline modification follows the recommendations made by the South African Guidelines Excellence (SAGE) project (Dizon et al. 2016). The process of guideline development is reported in detail later in this section.

Development of high quality and rigorously developed guidelines is a time-, labour- and cost-intensive process (Davino-Ramaya et al. 2012; Dizon et al. 2016; McCaul et al. 2018), often exceeding available resources in low-middle-income-countries (Dizon et al. 2016). In these contexts, guideline modification (adoption, adaptation or contextualisation) of existing high-quality guidelines provides a viable alternative (Dizon et al. 2016) that additionally maximises applicability to the local context, target population and target users.

#### Setting

The guideline is intended for use in a tertiary and academic public children’s hospital in South Africa, a low-middle income country with limited health resources (Argent et al. 2014; Rothe, Schlaich & Thompson 2013) and known health inequalities (Ataguba, Akazili & McIntyre 2011). Recommendations were made primarily with this facility in mind, although it was intended that the guideline should be potentially relevant to other tertiary, central or district hospitals in South Africa, and other low-middle income settings.

#### Population

A Population-Concept-Context (P-C-C) approach (Peters et al. 2017) was used to define the scope of this guideline as shown in Table 1.

**TABLE 1:** P-C-C defined search strategy.

P-C-C	Definition and search strategy
Population	Hospitalised children between the ages of 4 months to 13 years.
	*Include* Any age. Any patient acuity. *Exclude* Primary diagnosis of sleep disorder. Unstable or critical condition
Concept	Any guideline or recommendation on nonpharmacological sleep promotion in hospital.
Context	Paediatric wards in lower resourced hospital in South Africa, excluding hyper-acute care settings, such as high care and intensive care.
	*Include* Any in-patient healthcare facility. Any geographical setting. *Exclude* Out-patient and non-hospital care facilities.
Types of evidence sources	Practice guidelines and protocols. Detailed descriptions of guideline and protocol development process.
Date range	From 01 January 2010 to 17 February 2020
Language	Restricted to English, Afrikaans and Dutch

The majority of children admitted to the hospital are from households with very low incomes (Groenendijk et al. 2016) and present with chronic and or complex medical-, surgical- or medical-surgical conditions. An age range of 4 months to 13 years was specified for this guideline, because children in this range have relatively homogenous sleeping patterns (Claustrat, Brun & Chazot 2005). This age range also matches the age ranges treated at the target facility (Isaacs-Long, Myer & Zar 2017).

End users of the guideline were envisaged to be bedside nurses in conjunction with members of the wider healthcare team and in collaboration with the child’s bedside carer (most often the child’s mother) and staff. Involving mothers was crucial to the continuity of sleep for the child as sleep practices are known to vary by culture (National Research Council & Institute of Medicine 2000) and hospital (North et al. 2020).

#### Process of guideline development

**Step 1: Guideline identification:** Following the six-step methodology outlined above, transparent, structured and replicable searches were carried out with the objective of identifying existing guidelines or protocols on non-pharmacological sleep promotion in hospitalised children. Bibliographic database searching was conducted in PubMed, in line with the scope of this project. Manual searches of websites, including the International Paediatric Sleep Association and the South African Department of Health as well as websites known to offer accessible paediatric guidelines online, including The Royal Children’s Hospital Melbourne (Australia) and the website of the National Institute for Clinical Excellence (NICE United Kingdom [UK]), were performed to identify relevant guidance or policy documents. Keywords and MeSH terms included sleep, controlled environment, care, quiet (time), hospital, sleep and guideline or protocol. The search strategy was constructed using the P-C-C approach to aid definition of search terms and inclusion and exclusion criteria as summarised in Table 1. A specialist librarian was consulted. Table 1 demonstrates that search terms were broadened beyond the P-C-C defined scope of this guideline, allowing wider catchment of potential sources. Eligibility criteria included high quality guidelines that were developed for adults, in the expectation that they could be adapted. Guidelines were defined according to Kredo et al. (2016) as including statements of expected practice, benchmarks or standards enabling audit, comparison and potential improvement of practices or the presentation of structured recommendations about how to undertake particular tasks. Searches were carried out between 22 January 2020 and 17 February 2020. Results were screened using a tiered approach, narrowing the focus to P-C-C-relevant guidelines and protocols. High-quality studies and systematic reviews that were identified at the screening stage, but were not guidelines and protocols, were noted for reference at later stages of the process.

**Step 2: Appraisal of the existing guidelines:** Identified sources were then screened for scope and purpose, and rigour of development using Domains one and three from the AGREE II instrument (AGREE Next Steps Consortium 2017). The AGREE II instrument is an appraisal instrument that offers an objective measure to assess guideline quality and development (AGREE Next Steps Consortium 2017; The AGREE Collaboration 2003). The AGREE II instrument does not assess clinical content or the quality of evidence underpinning the recommendations (The AGREE Collaboration 2003).

Included items after screening were subjected to full AGREE II appraisal. Appraisals were conducted independently by a primary appraiser and two co-appraisers to reduce bias, adding rigour and reliability (The AGREE Collaboration 2003). All appraisers were children’s nurses enrolled in a Master’s degree and offered diverse African-centric perspectives. All had received training in using the AGREE II instrument. Decisions were reviewed by an independent nursing academic.

**Step 3: Compilation of the evidence base:** All existing recommendations were tabulated with the supporting rationale as recommended by Dizon et al. (2016). In the third step, after full appraisal, an adapted version of the NICE baseline assessment tool (NICE 2015) was used to consider each existing recommendation, determining local relevance and establishing the need for modification. Contextual insights were gained from literature and experience, and included factors such as transferability, cost and impact (Fischer et al. 2016; Van Achterberg, Schoonhoven & Grol 2008).

**Step 4: Modification of the recommendations:** The process of modification includes adoption (defined as utilising transferrable recommendations without alteration), contextualisation (making minor changes to recommendations to maximise impact and address local practice implications) and finally adaptation (requires altered wording, action and supplemental evidence) (Dizon et al. 2016).

Because the guideline was set in a South African nursing context, recommendations were considered with reference to the legal and regulatory frameworks governing the nurse’s scope of practice (SANC 2013:786) and the nurse’s regulation on acts or omissions in South Africa (SANC 2014:767).

**Step 5: Assigning levels of evidence and grades of recommendation:** As the fifth step, after meeting regulatory requirements, levels of evidence were assigned to evidence sources according to the approach exemplified by Xynos et al. (2016). Levels of evidence evaluate the quality of research and the anticipated impact of results (Burns, Rohrich & Chung 2011) from I (highest quality) to V (least robust) (Xynos et al. 2016). The rationale for each of the modified recommendations were derived from multiple evidence sources. Accordingly, aggregate levels of evidence were assigned to each modified recommendation. Aggregate scores prioritised the direction of results across studies (Rosenfeld et al. 2013). Relating the levels of evidence to practice, grades of recommendation estimate the effect of recommendation and accordingly imply adherence through specific recommendation wording (Rosenfeld et al. 2013). Grades of recommendation were influenced by considerations such as level of evidence, balance between benefit and harm, cost (Rosenfeld et al. 2013; Xynos et al. 2016) as well as values and preferences (Woolf et al. 2012).

**Step 6: End user guidan ce:** Finally, end user documentation was developed to improve guideline uptake amongst target users. A table of recommendations, with evidence-based rationales, was developed. To increase accessibility to evidence and optimise decision-making, recommendations were represented as a flowchart (see Figure 4) (Querido et al. 2018). Designing the process to start with safety and conclude with evaluation was considered likely to be a familiar process for nurses and was anticipated to increase guideline uptake amongst target users.

#### Consultation

To ensure maximum relevance of modified recommendations, consultations were conducted (Dizon et al. 2016). The content experts consulted included authors of the original guidelines selected for modification and four postgraduate-qualified children’s nurses familiar with the target setting. The process of consultation focused on obtaining feedback on draft recommendations and end user documentation, with suggestions for improvement. The structure for requesting comments was adapted from the NICE baseline assessment tool (NICE 2015) and related to priority, risk, local relevance, considerations of implementation (understandability and clarity), as well as evidence underpinning the proposed recommendations.

### Ethical considerations

This article followed all ethical standards for research without direct contact with human or animal subjects.

## Results

Bibliographic database searching (PubMed) resulted in the identification of 88 sources, whilst other methods identified 2345 sources (mostly grey literature). After de-duplication, a total of 2433 sources were screened by title and abstract or executive summary using the criteria described in Table 1. One guideline was identified (The Royal Children’s Hospital Melbourne 2015) as well as two published articles (Elliott & McKinley 2014; Knauert et al. 2018) which met the criteria for inclusion and were subjected to full text screening and searching of reference lists.

The authors of the above sources were contacted and provided additional information that led to the identification of Elliott (2012) and the Naptime Registered Nurse (RN) checklist (Knauert 2013), which were included as a subset of the Naptime protocol (Knauert et al. 2018).

AGREE II (AGREE Next Steps Consortium 2017) screening appraisals precluded further analysis of the Neonatal Sleep Maximisation guideline (The Royal Children’s Hospital Melbourne 2015), as the process of development was not described sufficiently to enable assessment. An email request for further information did not elicit a response.

At the end of this process, two sources (Elliott, McKinley & Tinker 2012; Knauert et al. 2018) were found suitable for modification.

*Rest and Sleep for the Intensive Care Patient* (Elliott et al. 2012) was a 22-page guideline developed by a nurse, containing 10 recommendations. The guideline was supplemented by a PhD thesis (Elliott 2011) and published research articles (Elliott, McKinley & Cistulli 2011; Elliott et al. 2013; Elliott & McKinley 2014), which provided further information about the process of development. The guideline outlined sleep promoting activities in an adult Intensive Care Unit in Sydney, Australia. The majority of recommended activities were applicable to nurses although the target audience was described as healthcare workers. Recommendations focussed on non-pharmacological measures, including noise reduction and cycled lighting (Elliott et al. 2012). The largest portion of the guideline was devoted to optimising the patient’s sleep, including pain control, optimising the patient’s normal circadian rhythm, providing a day-time rest period and ensuring optimal night-time sleep conditions (Elliott et al. 2012). This included cluster care, environmental interventions and manipulation of equipment such as ventilators (Elliott et al. 2012). Recording and monitoring tools such as the Richards Campbell Sleep Questionnaire (Richards, O’Sullivan & Phillips 2000) were recommended for use. One section focussed on pharmacological sleep promotion which was not included as it was beyond the scope of the guideline in development. The second considered source, *Naptime: an Overnight, Non-pharmacologic Intensive Care Unit Sleep Promotion Protocol* (Knauert et al. 2018), was published as an eight-page article in a peer-reviewed journal. The Naptime Protocol was developed as an overnight sleep promotion initiative for an adult medical Intensive Care Unit in the United States of America. The guideline envisaged a central role for the nurse in non-pharmacological sleep promotion based on a two tiered approach. Firstly, interventions targeted various levels of the organisation, for example, institutional, unit, bedside or direct care level. For the purposes of the guideline-in-development, focus was placed at the direct care level. The second tier was based on patient acuity, including timing of care, rescheduling certain activities and emphasising the importance of unhindered emergency care. In addition, an unpublished study document (Knauert 2013) provided by the author gave richer information on recommendations, for example, specifying the role of the nurse as a gatekeeper preventing undue disturbance during periods of rest (Knauert 2013).

**FIGURE 1:**
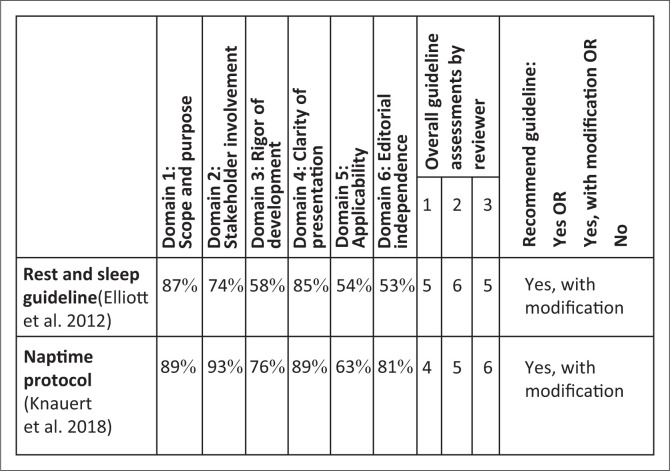
Domain scores of AGREE II full appraisal.

Figure 1 represents the full AGREE II appraisal of the Rest and Sleep guideline (Elliott et al. 2012) and the Naptime protocol (Knauert et al. 2018). The figure demonstrates higher domain-specific scores for the Naptime protocol (Knauert et al. 2018) and similar results for both sources’ overall guideline assessment. All appraisers independently supported the proceeding with the Rest and Sleep guideline (Elliott et al. 2012) and the Naptime protocol (Knauert et al. 2018) as guideline sources for modification.

**FIGURE 2:**
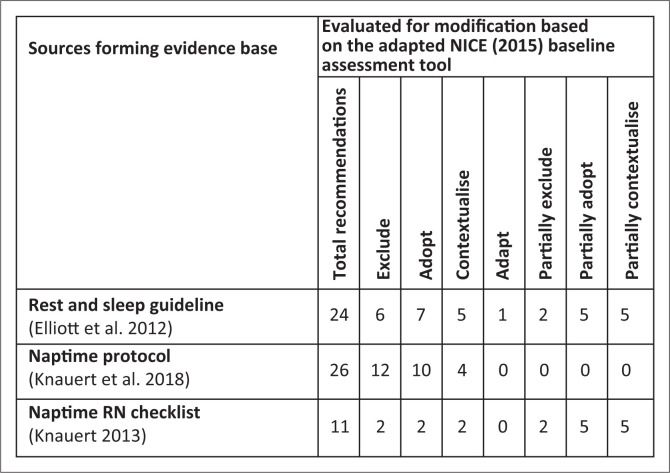
Evaluating recommendations in the evidence base.

Figure 2 presents the recommendations and outcomes of assessment using the adapted NICE (2015) baseline assessment. Decisions regarding adaptation or contextualisation of the recommendations are shown in Figure 2. In the development of the guideline, no recommendations were adopted without modification.

At the end of this process, 41 eligible recommendations were sequentially refined to six themes of recommendations, to reduce complexity and ensure that the new guideline be accessible to end users. These themes were: (1) safety and other special considerations, (2) collaboration with the bedside carer to promote individualised sleep, (3) coordination of the ward routine and (4) ward environment to improve sleep, (5) collaborating and communicating with staff and other persons to protect sleep and finally (6) performing a basic sleep assessment.

The majority of modifications to the recommendations involved contextualisation, mainly for differences in resources and target population. As an example, original recommendations about a visitor policy were contextualised to accommodate the expected presence of a mother or other bedside carer. A sleep assessment tool for children was identified (Owens & Dalzell 2005) and adapted, to replace the adult-specific tool (Richards et al. 2000) cited in the original guideline. The Regul8 tool, which focuses on supporting autonomic regulation in children, was included to guide nurses to ensure that children’s needs are met and they are well settled before periods of rest and sleep (Coetzee 2019).

**FIGURE 3:**
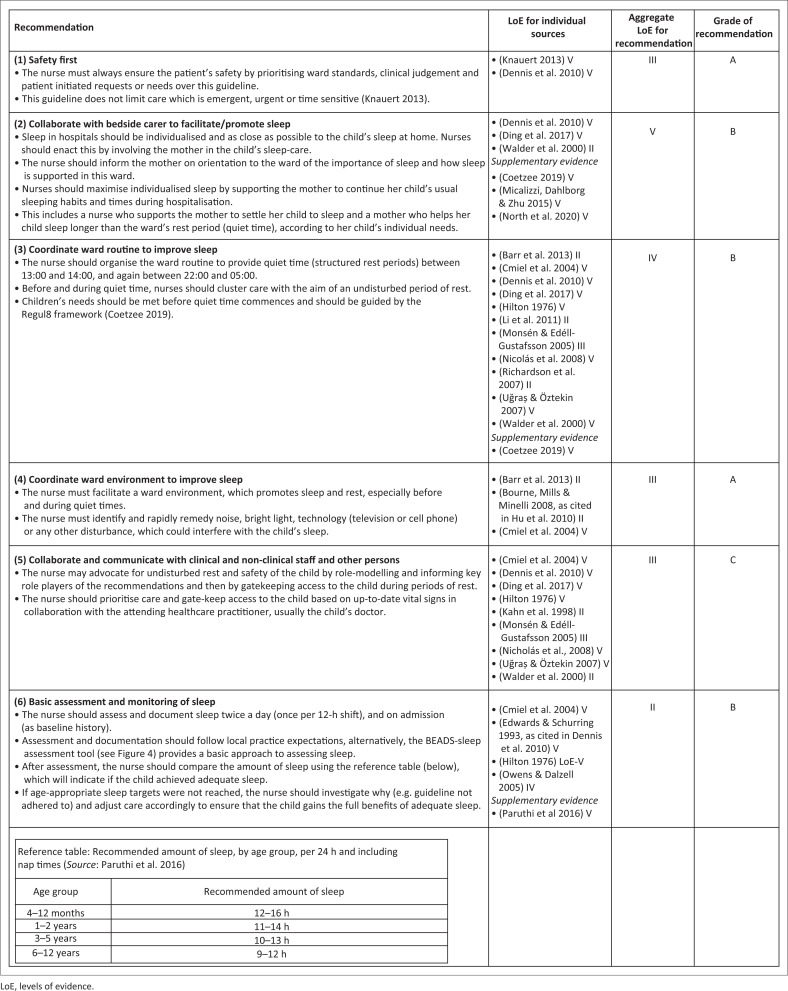
Recommendations with levels of evidence and grades of recommendation.

Next, 16 evidence sources, which underpinned original recommendations, and four supplementary evidence sources (Figure 3), which further supported modified recommendations, were assigned levels of evidence (LoE), as represented in Figure 3. Aggregated LoE and grades of recommendation are also displayed, arranged by final recommendations (see Figure 3).

Each recommendation theme was supported with additional rationales from the literature. These are included in the full version of the table of recommendations (see statement on data availability).

**FIGURE 4:**
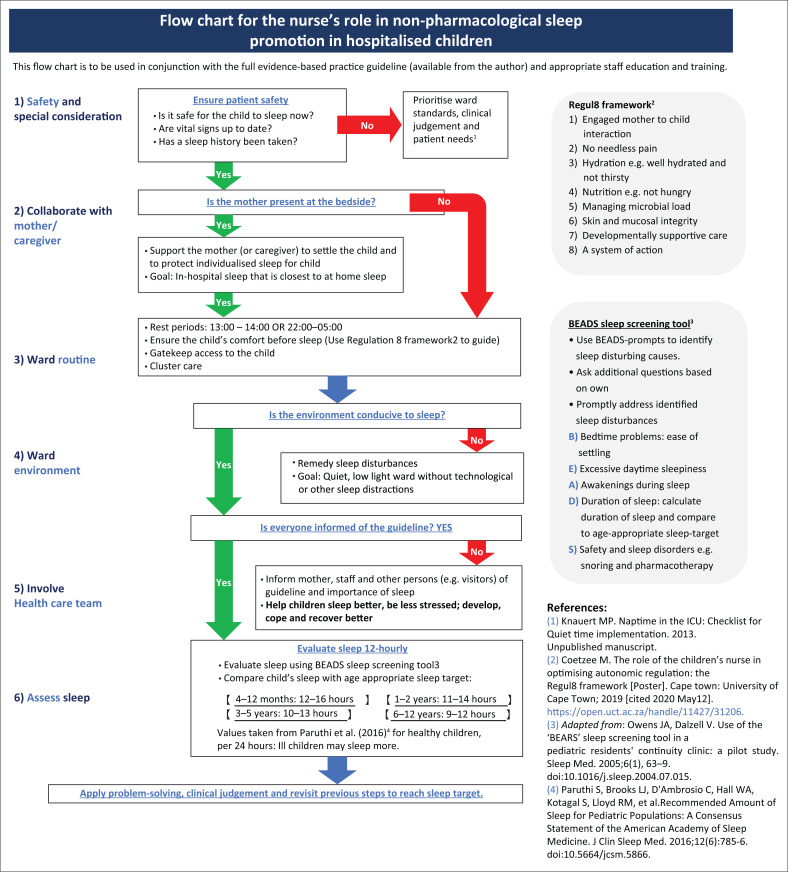
Flowchart summary of recommendations.

A flowchart summary of recommendations is presented in Figure 4 in poster format, for display in the ward.

The flowchart was incorporated into a poster summary together with an aide memoir: (1) safety first, (2) then a mother’s love; (3) next comes time; (4) low noise, dim light and calm. Pack the phone away, (5) collaborate with us; (6) then make sure sleep was enough.

## Discussion

### Key findings

Sleep is disrupted during paediatric hospitalisation (Bisogni et al. 2015; Kudchadkar, Aljohani & Punjabi 2014) through noise (Bevan et al. 2019) and care activities, for example, by taking vital signs (Peirce et al. 2018). Nurses, in particular, contribute to in-hospital sleep disruption (Bisogni et al. 2015). This guideline set out to improve sleep in hospitalised children as a holistic health approach by adding the benefits of good sleep to the child’s health trajectory, pursuing the objective of creating sleep-friendly ward environments and routines facilitated by nurses, and in partnership with mothers through six sets of recommendations as shown in Figure 3.

### Discussion of key findings

Healthcare workers prioritise ward routines above sleep to the extent that child healthcare staff have poor insight into the sleep disruptions associated with the care they provide (Peirce et al. 2018). Theoretical frameworks were identified that guide nurses to promote sleep in the context of infant development (Keys & Benzies 2018), but no age-appropriate guideline was identified. Relying on adult literature from high income settings required modification of recommendations.

It is quite possible to modify existing recommendations related to the six themes of: (1) Safety, (2) collaboration with the bedside carer to promote individualised sleep, coordination of the (3) ward routine and (4) ward environment to improve sleep, (5) involvement of the wider ward team and (6) sleep assessment. These recommendations and the supporting evidence-base are well-described in Figure 3 and an extended rationale is available as a supplementary file (refer to the ‘Data availability statement’). We, therefore, highlight two aspects of the evidence-base as it informed the modification process, firstly in relation to collaboration with the bedside carer, and secondly in relation to sleep assessment.

The second recommendation, concerning collaboration with the bedside carer, was adapted from original recommendations that encouraged family members and visitors to rest at home (Knauert et al. 2018), and for guideline users to provide settling procedures as close to the patient’s usual night-time routines and preparing the patient to sleep (Elliott et al. 2012; Knauert 2013; Knauert et al. 2018). Recommendations were, understandably, adult-centric and related to high income settings. For example, bed bells were to be placed close to patients and room-telephones to be unplugged (Knauert 2013), which are not conditions that apply to the local setting. Modification ensured that local resource constraints were reflected, including the purposeful involvement of mothers in the care of their hospitalised child. In-hospital, familial involvement maximises limited resources, decreases staff burnout and improves patient safety (Micalizzi et al. 2015). Families desire to be involved in their child’s hospital care (Bisogni et al. 2015; Micalizzi et al. 2015; North et al. 2020) and the intentional involvement of mothers and caregivers holds promise to promote sleep in hospitals by capitalising on nurse’s relational strengths (Keys & Benzies 2018). For the child and family, the benefits may extend beyond hospital discharge (Erondu et al. 2019). Nurses also benefit, for example, periods of rest for patients (also called quiet time), is found to lower nurse’s stress levels in a hyperacute setting (Riemser et al. 2015).

Turning to recommendation six (sleep assessment), we noted that children’s sleep needs differ in quantity by age, generally decreasing from infancy towards puberty (Grigg-Damberger 2016). When children are ill, they require additional sleep (Gamaldo et al. 2012) and interpretation of any sleep-assessment tool should take this into account. Accordingly, the Richards Campbell Sleep Questionnaire (Richards et al. 2000) used in the adult-centric Rest and Sleep guideline (Elliott et al. 2012) was not suitable for use in children as it required a level of understanding and fine motor skills beyond the ability of children in certain developmental stages. Alternative child-friendly sleep assessment tools were considered based on quantity and quality of sleep, complexity/brevity, ease of use, target audience and the intended purpose of use. The BEARS-sleep screening tool (Bedtime Issues, Excessive Daytime Sleepiness, Night Awakenings, Regularity and Duration of Sleep, Snoring) (Owens & Dalzell 2005) was found best suited but required adaptation to the local context of use. The BEARS-sleep screening tool (Owens & Dalzell 2005) was adapted (Lee & Ward 2005) to a brief and locally relevant BEADS-sleep screening tool (see Figure 4 and data availability statement).

The results of this process of guideline modification suggest that in the absence of existing guidelines specific to non-pharmacological sleep promotion for our intended child population and setting, it was nonetheless possible to modify existing guidelines and apply the associated evidence base to provide evidence-based recommendations to assist nurses in optimising the quantity and quality of sleep in hospitalised children, contributing to the longer-term goal of aiding healing and emotional well-being.

### Strengths and limitations

The recommendations are mainly underpinned by evidence from the literature that focussed largely on benefits and studies which were conducted in higher-income settings. This was mitigated by ensuring that the rationales for recommendations were informed by local peer review and international expert consultation. Comprehensive reporting of methodology and results has been followed to support transparency and enhance trust in the recommendations, highlighting the gaps in the current evidence base. Because this guideline has not yet been implemented and was based in a lower income setting, implementation may reveal considerations that were not identified through expert consultation or existing literature. Research in this regard would be valuable, including validation of an in-patient sleep screening tool such as the adapted BEADS sleep screening tool.

### Implications or recommendations

Nurses need to assess the relevance and suitability of the guideline prior to implementation in their own practice settings, in conjunction with the wider multidisciplinary healthcare team and the leadership of the facility. Implementation should be accompanied by a baseline assessment and evaluation. The guideline should be updated in five years, or sooner based on emerging evidence and or outcomes of the guideline (Rosenfeld et al. 2013). Future updates of the guideline should therefore remedy barriers, promote facilitators and add to the evidence base.

## Conclusion

Hospitalisation challenges children’s autonomic regulatory abilities. Dysregulation is amplified through poor sleep, a consequence of hospitalisation where care is prioritised above sleep. Nurses are often unaware of the disruption they contribute to children’s (loss of) sleep during hospitalisation. However, nurses are ideally positioned to capitalise on time spent in hospital to aid children’s sleep through engagement and partnering with families, to promote health and enact their role as patient advocates. The guideline described throughout this article seeks to guide nurses to actively engage in sleep promotion for the hospitalised child. This evidence-based African-centric approach of partnering with families during hospitalisation exemplifies collaborative healthcare and holds the promise of benefits that extend beyond discharge home in resource-constrained contexts.
